# Karyotype stability of human umbilical cord-derived mesenchymal stem cells during *in vitro* culture

**DOI:** 10.3892/etm.2014.1977

**Published:** 2014-09-18

**Authors:** ZHONG-BAO RUAN, LI ZHU, YI-GANG YIN, GE-CAI CHEN

**Affiliations:** Department of Cardiology, Taizhou People’s Hospital, Taizhou, Jiangsu 225300, P.R. China

**Keywords:** human umbilical cord-derived mesenchymal stem cells, serial passage, karyotyping

## Abstract

The aim of this study was to investigate whether the chromosomes of human umbilical cord-derived mesenchymal stem cells (hUCMSCs) change following *in vitro* culture for several generations. In the present study, umbilical cords from two healthy infants following cesarean delivery were collected aseptically and hUCMSCs were isolated by digestion with collagenase and trypsin, and then cultured *in vitro*. hUCMSCs with fibroblastic morphology were presented from the human umbilical cord tissue after 7 days of adherent culture. When cultured for 6 passages *in vitro*, the hUCMSCs maintained a stable spindle-shaped morphology. Cells reached the logarithmic growth phase after 3–4 days of culture. In addition, CD13, CD29, CD44, CD90 and CD105 were highly expressed in generations P3-P6. The expression of CD31, CD34, CD45 and HLA-DR was negative. Furthermore, karyotype analysis revealed a normal diploid karyotype with 46 chromosomes and no abnormal changes were found in chromosome structure. These findings suggest that when cultured for 6 passages *in vitro*, hUCMSCs maintain a stable immunophenotype and chromosome structure, which provides an experimental basis for the safety of hUCMSC cytotherapy.

## Introduction

Mesenchymal stem cells (MSCs) possess the capacity for self-renewal and multi-directional differentiation, and have several characteristics, including multi-lineage differentiation potential, hematopoietic support and stem cell implantation promotion, immune regulation and self-renewal, which make them a promising source for cell therapy in numerous diseases (1–3). Since MSCs are rarely found in bone marrow or fetal tissues, the isolation and expansion of human MSCs is crucial for their clinical application. Human umbilical cord-derived mesenchymal stem cells (hUCMSCs) are readily available, abundant, rich in content and able to differentiate into a variety of types of cells. As a result, they have been the focus of considerable attention. However, whether the chromosomes of hUCMSCs change following culture *in vitro* remains unclear and controversial (4). Therefore, in the present study hUCMSCs were isolated and cultured *in vitro*, and the alteration in karyotypes of the hUCMSCs subjected to serial passage *in vitro* was determined by investigating the karyotypes of generations of P0 and P3-P6 and analysis with the G-banding technique.

## Materials and methods

### Materials

The materials used included DMEM/F12 medium (NVF0274; Gibco-BRL, Gaithersburg, MD, USA), fetal bovine serum (FBS; 10057; Excell Bio, Shanghai, China), collagenase II (Gibco-BRL), trypsin (Gibco-BRL), cell culture box (Nunc; Thermo Fisher Scientific, Waltham, MA, USA), flow cytometer (BD Accuri C6; BD Biosciences, Franklin Lakes, NJ, USA), inverted optical microscope (IX70-S8F; Olympus, Tokyo, Japan), mouse anti-human CD13, CD29, CD31, CD34, CD44, CD45, CD90, CD105 and HLA-DR antibodies (BD Biosciences), karyotyping system (CytoVision version 7.2; Leica, Mannheim, Germany) and a medium kit for chromosome culture (RPMI; Gibco-BRL, Grand Island, NY, USA). Colchicine, glacial acetic acid and Giemsa stain (Sigma-Aldrich, St. Louis, MO, USA) were also used.

### Isolation and culture of hUCMSCs

Umbilical cords were obtained following normal or cesarean term deliveries from two healthy infants (one male and one female) at Taizhou People’s Hospital (Taizhou, China) in January 2013. Written informed consent was obtained from the mothers and the experimental procedures were approved by the Ethics Committee of Taizhou People’s Hospital. The umbilical cords were cannulated and washed three times with phosphate-buffered saline to remove blood clots. Then the umbilical cords were cut into 2–3 cm pieces and opened with a scalpel. The Wharton’s jelly was scratched out and the vessels were removed. Thereafter, the Wharton’s jelly was incubated in collagenase/DMEM solution for 24 h. Following centrifugation for 10 min, the cell pellet was resuspended and incubated in trypsin at 37°C for 30 min. Finally, the cell pellet was resuspended in DMEM/F12 medium containing 10% FBS and cells were seeded in a T75 culture flask. The cells were passaged at 1×10^4^–0.4×10^6^cells/cm^2^, and were continuously cultivated for six passages. Morphological changes were assessed by observing the cells under an inverted optical microscope.

### Detection of surface markers of hUCMSCs using flow cytometry

Following the third passage (P3), the cells were washed twice with PBS, and then digested with a 1:1 mixture of trypsin (2.5 g/l) and EDTA (0.2 g/l). A suspension of 1×10^10^ cells/l was obtained by washing with PBS containing bovine serum albumin (20 g/l). A total of 100 μl cell suspension was added to each Eppendorf tube. Fluorescently labeled anti-human-antibodies [PE-CD13, PE-CD29, APC-CD90, PE-CD105, PE-CD31, PE-CD34, FITC-CD44, FITC-CD45 and FITC-HLA-DR] were added in 20 μl. To the control group was added immunoglobulin G (IgG; isotype control). The cells were incubated for 30 min at 4°C in the dark, washed twice with PBS, then 200 μl paraformaldehyde (10 g/l) was added to each tube. Flow cytometry was used to detect the surface markers of hUCMSCs.

### Cell proliferation test

Proliferation curves were determined using the MTT method. Each passage of hUCMSCs was respectively seeded in a 96-well plate; 200 μl cell suspension with a density of 2×10^4^/ml was added to each well. The cells were incubated for 72 h and 10 μl MTT reagent (Sigma-Aldrich) was then added to each well. The cells were incubated for a further 4 h, the medium was removed and 200 μl DMSO was added to each well to dissolve the formazan. The plates were shaken for 15 min at 10 × g. The number of the cells per well was counted every day for 8 successive days and used to construct a proliferation curve for the hUCMSCs.

### Karyotyping of generations

According to the result of the flow cytometric analysis and cell proliferation test, karyotypes were analyzed in hUCMSCs from the first passage that was isolated and cultured *in vitro* using the chromosome G-banding technique. The cell suspension (300 μl) with a density of 2×10^4^/ml was added to a 10-ml culture bottle, then 100 μl colchicine with a concentration of 40 μg/ml was added to the culture bottle. The cells were incubated for 4 h in a carbon dioxide incubator and the adherent cells were removed and placed into a 15-ml centrifuge tube. Following centrifugation for 8 min at 182 × g, the culture medium containing colchicine was removed and 4 ml 0.075% KC1 was added. Following incubation at 37°C for 5 min, 2 ml Carnoy’s solution (3:1 v/v absolute ethanol: glacial acetic acid) was added and the cells were maintained in culture at 37°C for 5 min. The cells were centrifuged for a further 8 min at 182 × g, the culture medium was removed and 4 ml Carnoy’s fixative was added. The cells were incubated and centrifuged again, which was repeated twice. The cell pellet was resuspended in DMEM/F12 medium containing 10% FBS and aliquots of the suspension were dropped onto slides. After 48 h at room temperature, the slides were placed in a slide drier 75°C for 4 h. Giemsa staining was then performed for 15 min and karyotypes of generations were analyzed using the G-banding technique.

## Results

### Morphology of hUCMSCs

**Figure 1:**
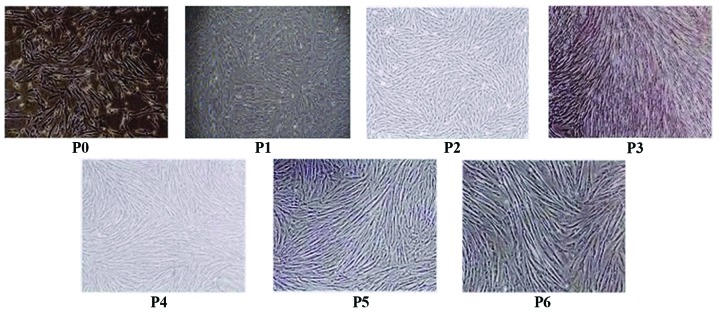
Morphology of human umbilical cord-derived mesenchymal stem cells (inverted microscope; magnification, ×40). P, passage.

After 7 days of adherent culture, hUCMSCs with fibroblastic morphology were presented from the human umbilical cord tissue. Following serial passage, the cells grew and proliferated quickly. When cultured for 6 passages *in vitro*, hUCMSCs maintained a stable spindle-shaped morphology (Fig. 1).

### Immunophenotype

**Figure 2:**
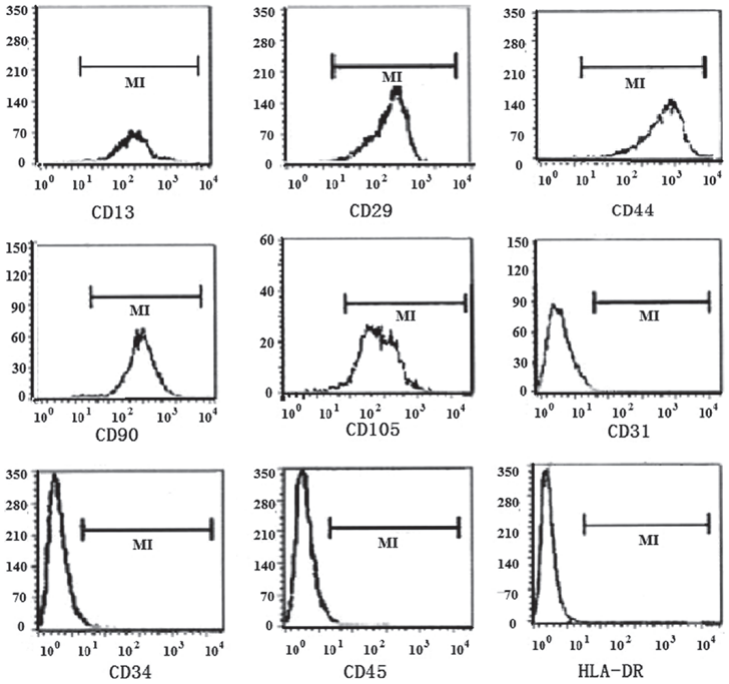
Immunophenotype results of human umbilical cord-derived mesenchymal stem cells.

The results from the flow cytometric analysis revealed that standardized culture of hUCMSCs *in vitro* resulted in the stable expression of surface markers following serial passage. CD13, CD29, CD44, CD90 and CD105 were highly expressed at a level of >95% on the surface of hUCMSCs, but the expression of CD31, CD34, CD45 and HLA-DR was negative and <2% (Table I, Fig. 2).

### Proliferation of hUCMSCs

**Figure 3:**
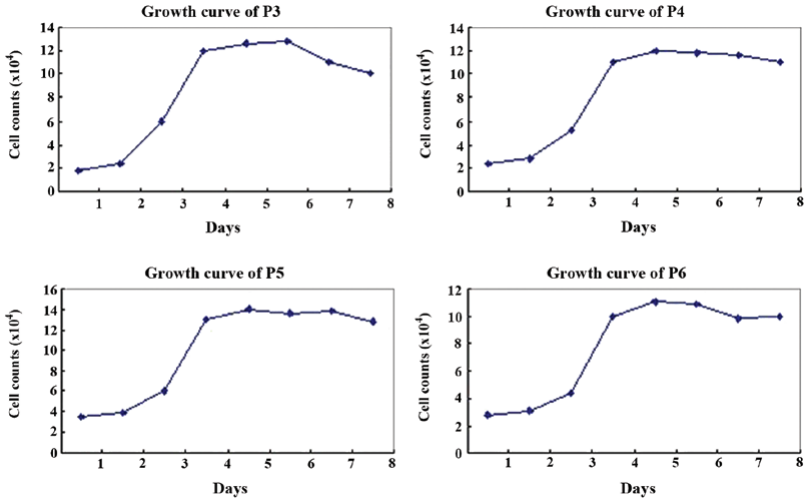
Growth curves of human umbilical cord derived mesenchymal stem cells. P, passage.

Each passage of hUCMSCs presented a typical S-like proliferation curve. Cells were in a slow growth period at 1–2 days of culture, but reached a logarithmic growth phase at 3–4 days and then a plateau phase at 5–7 days. After 8 days of culture, the cells were observed to have diminished proliferation potency (Fig. 3).

### Karyotype analysis of hUCMSCs

**Figure 4:**
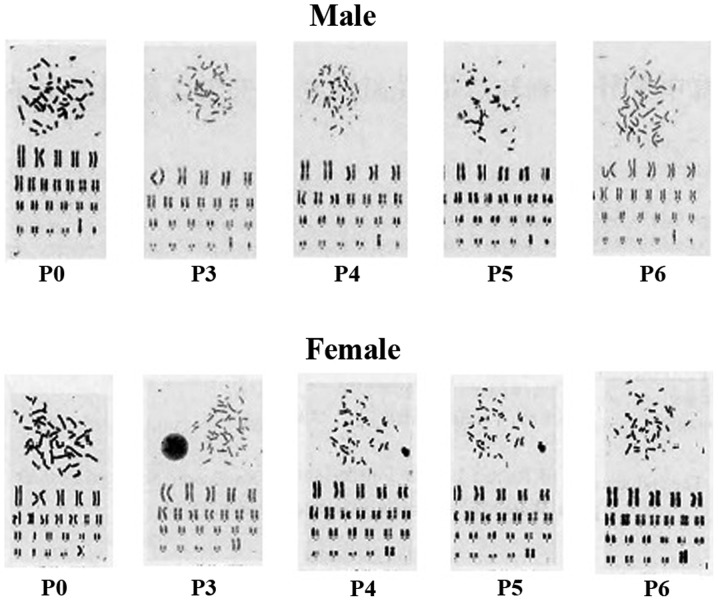
Karyotype analisis of human umbilical cord derived mesenchymal stem cells. P, passage.

Following treatment with colchicine, P3-P6 of hUCMSCs were arrested in the metaphase stage. A normal diploid karyotype with 46 chromosomes and no abnormal changes in chromosome structure was observed by the analysis of 30 metaphase cells (Fig. 4). However, abnormal morphology was observed in the seventh and eighth passages, and the ratio of non-hUCMSCs also increased in these passages.

## Discussion

MSCs offer a lot of promise for the development of novel alternative cell-based therapies. These unique cells possess two major features: their ability for self-renewal and differentiation potential. At present, MSCs are primarily obtained from bone marrow. However, bone marrow-derived MSCs are easily contaminated by viruses and have a significantly decreased capacity for proliferation and differentiation with the aging of the donor. In addition, as there are a variety of ethical and legal issues, their application is restrained (5,6). Umbilical cord blood (UCB) is a source of additional stem cells for experimental and potentially clinical uses. However, the presence of MSCs in UCB is controversial (7). Human umbilical cord, a connecting tissue of extraembryonic origin lying between the mother and fetus, consists of two arteries, one vein, inter-vessel connective tissue and umbilical epithelium. The connective tissue, also referred as Wharton’s jelly, is composed of a sponge-like structure woven by collagen fibers, proteoglycan and embedded stromal cells. The umbilical cord is a promising source of MSCs due to its wide availability, differentiation potential and lack of ethical concerns. Compared with bone marrow MSCs, hUCMSCs are easier to isolate and expand, and so they would be an advantageous, novel source of adult MSCs. A number of studies have demonstrated that hUCMSCs may have an important role in the application and experimental research of adult human MSCs (8,9).

Due to differences between culture systems *in vitro* and conditions *in vivo*, whether MSCs maintain a stable chromosome structure following serial passage and proliferation is an important index of clinical safety. Numerous studies have found that an abnormal chromosome structure is positively correlated with the incidence of tumors (10,11). The results of investigations of the karyotype stability of MSCs during a long-term culture *in vitro* remain inconsistent. It has been demonstrated that MSCs passaged >10 times have a chromosome analysis that is normal (4,12); however, other studies have found that karyotype variation in MSCs may occur following long-term culture *in vitro* and have tumorigenicity in nude mice (13–15). Although hUMSCs are a desirable source of cells for use in tissue repair and regeneration engineering, few studies have addressed the safety of hUMSCs that have been subjected to long-term culture *in vitro*.

In the present study, by digestion with collagenase and trypsin, a large number of adherent hUMSCs were harvested within a short time period. The results showed that following resuscitation, these cells maintain high cell viability, and could be cultured for a long time, enabling large numbers of cells to be harvested. The results from the flow cytometric analysis demonstrated that the hUCMSCs harvested in the present study maintained their phenotypes following long-term *in vitro* proliferation and serial passage. CD13, CD29, CD44, CD90 and CD105 were highly expressed on >95% of the surface of hUCMSCs; however, the expression of CD31,CD34, CD45 and HLA-DR was negative and <2%, which is consistent with a previous study (16). The results from the proliferation test demonstrated that each passage of hUCMSCs presented a typical S-like proliferation curve. Cells were in a logarithmic growth phase after 3–4 days of culture. According to the results of proliferation test, the hUCMSCs of P3-P6 were selected, which were then cultured for 3–4 days for karyotyping. The karyotype analysis showed a normal diploid karyotype with 46 chromosomes and no abnormal changes were observed in chromosome structure in the hUCMSCs of P3-P6 following treatment with colchicine. In the present study, it was found that there was abnormal morphology in the seventh and eighth passages and the ratio of non-hUCMSCs was also increased in these passages.

In conclusion, the present study demonstrated that hUCMSCs maintain a stable immunophenotype and chromosome structure when cultured for 6 passages *in vitro*, which provides an experimental basis for the safety of hUCMSC cytotherapy.

There were a number of limitations in the present study. The number of the subjects in this study was small, which may have affected the reliability of the results. The karyotype stability of hUCMSCs was not evaluated following long-term culture. It remains to be determined whether the chromosomes of hUCMSCs change following *in vitro* culture for larger numbers of generations and over a longer period of time in future studies.

## Figures and Tables

**Table I:** Immunophenotype of human umbilical cord-derived mesenchymal stem cells detected using flow cytometry (%).

Passage	CD13	CD29	CD44	CD90	CD105	CD31	CD34	CD45	HLA-DR
P1	96.70	98.30	95.60	99.20	97.10	0.69	1.24	0.63	0.18
P2	97.30	96.40	96.10	99.30	95.90	0.18	0.79	0.51	0.24
P3	99.20	96.90	95.40	99.80	95.10	0.71	1.00	0.24	0.56
P4	95.40	97.20	97.20	99.60	96.40	0.62	0.68	0.72	0.05
P5	98.70	98.60	96.30	98.70	96.70	0.34	0.92	0.29	0.25
P6	96.50	95.10	95.80	99.10	98.10	0.59	0.75	0.46	0.36
